# Draft genome assembly of *Lacticaseibacillus paracasei* MSR2 isolated from Bangladesh

**DOI:** 10.1128/mra.01314-24

**Published:** 2025-04-30

**Authors:** Md. Shahedur Rahman, Ashikuzzaman Antor, Mahadi Hasan Sojol, Md. Monir Hossen, Mahadi Hasan, Rafi Hasnat Sarker, Mohammad Abu Hena Mostofa Jamal

**Affiliations:** 1 Department of Genetic Engineering and Biotechnology, Jashore University of Science and Technology, Jashore, Bangladesh; 2 Bioinformatics and Microbial Biotechnology Laboratory, Jashore University of Science and Technology, Jashore, Bangladesh; 3 Department of Biotechnology and Genetic Engineering, Islamic University 247297, Kushtia, Bangladesh; 4 Laboratory of Medical and Environmental Biotechnology, Islamic University, Kushtia, Bangladesh; University of Maryland School of Medicine, Baltimore, Maryland, USA

**Keywords:** WGS, Genomics, *Lacticaseibacillus paracasei* MSR2

## Abstract

This study reports the draft genome sequence of *Lacticaseibacillus paracasei* MSR2, a strain isolated from yogurt in Bangladesh in 2024. The genome consists of a 3,085,195 base pair chromosome assembled into 132 contigs, encoding 2,903 predicted proteins.

## ANNOUNCEMENTS

*Lacticaseibacillus paracasei,* a probiotic commonly present in dairy products, supports host health by offering numerous benefits (1, 2). *L. paracasei* has also been reported to exhibit antifungal and antibacterial activities (3). This study presents the draft genome assembly of *L. paracasei* MSR2.

A yogurt sample was collected from Kishoreganj, Bangladesh, in February 2024. The sample was spread onto De Man–Rogosa–Sharpe (MRS) agar (HiMedia, India) and incubated overnight at 37°C. A single colony from the pure culture was inoculated into MRS broth and incubated overnight at 37°C with shaking at 120 rpm. One milliliter of broth was used for genomic DNA extraction. DNA extraction was carried out using the Qiagen DNeasy Blood & Tissue Mini Kit (250) following the manufacturer’s protocol. After cell lysis, the sample was centrifuged at 14,000 rpm for 5 minutes to remove cellular debris. The DNA was further purified using the CDC PULSENET Total DNA Extraction protocol using the same kit. DNA quality was assessed using a Nanodrop spectrophotometer and a Qubit 4.0 Fluorometer. For Qubit measurements, a working solution was prepared by mixing Qubit Reagent and Qubit Buffer in a 1:200 ratio. Whole-genome sequencing was performed at the Genomic Centre, icddr’b, Mohakhali, Dhaka, Bangladesh. The DNA fragments were size selected using AMPure XP beads to isolate those within the desired range, typically 200–300 base pairs (bp) for standard Illumina sequencing libraries. The library for sequencing was prepared using the Illumina DNA Prep Reagent Kit and an automated liquid handler (epMotion 5075) for tagging the adapters with DNA fragments. After library preparation, sequencing was carried out using the Illumina MiSeq platform with paired-end 2 × 150 bp reads following the standard protocol provided by the manufacturer. Illumina’s bcl2fastq software version 2.20.0 (https://support.illumina.com/sequencing/sequencing_software/bcl2fastq-conversion-software.html) was utilized with default parameters to demultiplex the raw reads and convert them into FASTQ format. For further downstream analysis, raw paired-end reads from the MiSeq platform were used.

FASTQC version 0.9.1 (www.bioinformatics.bbsrc.ac.uk/projects/fastqc) was employed to assess the quality of the raw data (4). Sickle version 1.3 tool was used to trim the paired-end sequence, where the trimming length was set to 20 (5). For assembly, Spades version 4.0.0 was applied with default parameters (6). The assembly was polished using Pilon version 1.24 (7). The species was identified using a whole genome-based taxonomic analysis in the Type (Strain) Genome Server (https://tygs.dsmz.de). Default parameters were used for all software unless otherwise specified.

The genome of *L. paracasei* MSR2 is 3,085,195 bp long with a GC content of 46.5%. The assembly contains 132 contigs with a coverage of 111.8×. A total of 3,019 genes were predicted, of which 2,852 are protein-coding genes. The details of genomic features are presented in Table 1.

**TABLE 1:** Genomic features of *Lacticaseibacillus paracasei* MSR2

Description of source	
Location	Kishoreganj, Bangladesh
Time	2024
Type	Yogurt
Sequencing summary	
Coverage	111.8×
Total bases	3,074,103
GC content	46.27%
Reads	865,257
Assembly report	
Contigs	132
GC content	46.5%
Contig *L*_50_	10
Genome length	3.1 Mb
Contig *N*_50_	98.4 kb
Annotation report	
Genes	3,079
CDs	2,903
CDs (without protein)	105
ncRNAs	3
tRNA	58

## Data Availability

This Whole Genome Shotgun project has been deposited in GenBank under accession no. JBJNKY000000000.1. The raw reads are available in SRA under accession number SRR31790714. The version described in this paper is the first version, JBJNKY000000000.1. The data have been deposited in GenBank under BioSample number SAMN45146297 and BioProject number PRJNA1193548. The genome assembly is available under the accession ASM4580884v1.
